# Healthy Sleep Associated With Lower Risk of Hypertension Regardless of Genetic Risk: A Population-Based Cohort Study

**DOI:** 10.3389/fcvm.2021.769130

**Published:** 2021-11-18

**Authors:** Zhi-Hao Li, Qing-Mei Huang, Xiang Gao, Vincent C. H. Chung, Pei-Dong Zhang, Dong Shen, Xi-Ru Zhang, Wen-Fang Zhong, Dan Liu, Pei-Liang Chen, Qing Chen, Miao-Chun Cai, Xin Cheng, Hai-Lian Yang, Wei-Qi Song, Xian-Bo Wu, Virginia Byers Kraus, Chen Mao

**Affiliations:** ^1^Department of Epidemiology, School of Public Health, Southern Medical University, Guangzhou, China; ^2^Department of Nutritional Sciences, Pennsylvania State University, University Park, PA, United States; ^3^Jockey Club School of Public Health and Primary Care, The Chinese University of Hong Kong, Shatin, Hong Kong SAR, China; ^4^Division of Rheumatology, Department of Medicine, Duke University School of Medicine, Duke Molecular Physiology Institute, Durham, NC, United States; ^5^Division of Laboratory Medicine, Microbiome Medicine Center, Zhujiang Hospital, Southern Medical University, Guangzhou, China

**Keywords:** sleep, genetic risk, hypertension, cohort study, epidemiology

## Abstract

**Background:** Hypertension is a leading contributor to the global burden of disease and to mortality. The combined effects of sleep factors on the risk of hypertension are unclear. We aimed to evaluate the effect of combined sleep factors on the risk of hypertension and to explore whether this association is independent of genetic risk.

**Methods:** This population-based prospective cohort study included 170,378 participants from the UK Biobank study. We conducted a healthy sleep score based on a combination of major five sleep factors and a genetic risk score based on 118 risk variants. Cox proportional hazard regression models were used to estimate hazard ratios (HRs) and 95% confidence intervals (CIs).

**Results:** A total of 170,378 participants were included. Compared to participants with a healthy sleep score of 0–1, those with healthy sleep scores of 2 (HR, 0.90; 95% CI, 0.83–0.98), 3 (HR, 0.81; 95% CI, 0.75–0.88), 4 (HR, 0.74; 95% CI, 0.68–0.81), or 5 (HR, 0.67; 95% CI, 0.59–0.77) had increasingly lower risks of hypertension (*P* for trend <0.001). Participants with high genetic risk and an unfavorable sleep pattern had a 1.80-fold greater risk of hypertension than participants with low genetic risk and a favorable sleep pattern. The association between sleep patterns and hypertension persisted in subgroup analysis, stratified by the genetic risk. Nearly 18.2% of hypertension events in this cohort could be attributed to unfavorable sleep pattern.

**Conclusions:** Favorable sleep pattern was associated with a low risk of hypertension, regardless of genetic risk. These findings highlight the potential of sleep interventions to reduce risk of hypertension across entire populations.

## Introduction

Hypertension is a leading contributor to the global burden of disease and to mortality (1). It is a complex disease driven by both environmental and genetic factors (2–6). Sleep factor is an important modifiable risk factor for hypertension (7, 8). A rich body of evidence shows that healthy sleep factors, including no excessive daytime sleepiness (9), adequate sleep duration (10, 11), no insomnia (12, 13), no snoring (14, 15), and early chronotype (16), were associated with low risk of hypertension. Although those sleep factors have been independently correlated to increased risk of hypertension, a combination of sleep factors may have synergistic effects as these sleep factors are highly associated with each other (17–20). Bathgate et al. (21) have assessed sleep factors jointly and indicated the highest risk of hypertension in association with a joint effect of short sleep duration (<6 h) with insomnia. Therefore, a composite variable may better aid investigation of how sleep factors act synergistically to affect hypertension risk. However, few studies have investigated the combined impact of sleep factors, let alone all the aforementioned sleep factors jointly, on hypertension risk.

Early evidence supporting a role for genetics in the risk of hypertension came from twin studies and the Framingham Study (5, 22–24). Further evidence has emerged from genome-wide association studies (GWASs), which have identified genetic variants associated with the risk of hypertension (2, 25). These risk alleles, when aggregated into a polygenic risk score, are predictive of incident hypertension and provide a quantitative measure of the genetic risk for hypertension. It might be hypothesized that adhering to a healthy sleep pattern could attenuate the effect of genetics on the risk of hypertension. A previous study on cerebrovascular disease, a condition closely related to hypertension, found a statistically significant role of the interplay between sleep factors and genetics in the risk of cerebrovascular disease (17). However, whether a healthy sleep pattern, which integrates several modifiable sleep factors, can modify the effect of genetic predisposition on hypertension remains less certain; moreover, no study to date has investigated the risk of hypertension with regard to sleep patterns and genetics.

Therefore, in a large population-based cohort study, we prospectively investigated the association of a healthy sleep score based on a combination of major sleep factors with the risk of incident hypertension. We further explored whether the association between sleep pattern and the risk of incident hypertension was independent of genetic risk.

## Methods

### Study Population

The UK Biobank study is a large, population-based prospective cohort study that recruited >500,000 individuals aged between 40 and 70 years from across the UK (Scotland, England, and Wales) between 2006 and 2010. The population and design of the UK Biobank study have been described in detail in previous reports (26–29). The study collected extensive data, including demographic, health, and lifestyle (e.g., sleep factors) data, from questionnaires, interviews, physical measurements, and health records. Blood samples were also obtained and used for genotyping (30). Approval for this research was obtained from the North West Multicenter Research Ethics Committee (11/NW/0382), and all participants provided informed consent.

In this study, after excluding participants with hypertension or cardiovascular diseases at baseline, those with missing data for any of the five sleep factors, or those without genetic data, 170,378 participants were finally included in the present analyses. A flowchart depicting the selection of the study participants is presented in Supplementary Figure 1.

### Measure

#### Healthy Sleep Score and Sleep Pattern

The UK Biobank participants completed a self-reported touchscreen questionnaire on their usual sleep factors. To investigate the association between the combination of sleep factors and hypertension, we constructed a healthy sleep score based on the recommendations regarding five potentially modifiable sleep factors, including daytime sleepiness, sleep duration, insomnia, snoring, and chronotype, on the basis of previous studies (13, 17, 18). The methods of assessment of the sleep factors is described in Appendix. We dichotomized each sleep factor based on previous knowledge (17). Participants were assigned one point for each of five low-risk sleep factors defined as follows: sleep 7–9 h per day, no insomnia (“never/rarely” having insomnia symptoms), no snoring, early chronotype (“morning” or “morning more than evening” person), and no frequent daytime sleepiness (“never/rarely” or “sometimes”). The points for the five sleep factors were summed to obtain the healthy sleep score, which ranged from 0 (least healthy) to 5 (most healthy), and was subsequently categorized as a favorable (score of 4 or 5), intermediate (score of 2 or 3), or unfavorable (score of 0 or 1) sleep pattern, as described previously (17).

A weighted standardized healthy sleep score was then derived based on the five sleep factors with the following equation: weighted sleep score = (β_1_×sleep factor1 + β_2_×sleep factor2 +…+ β_5_×sleep factor5) × (5/sum of the β coefficients). This weighted sleep score also ranged from 0 to 5 points but took into account the magnitudes of the adjusted risk for each behavior in each sleep pattern and in the combination of the five sleep factors (17).

### Genotyping and Polygenic Risk Score

The genotyping process in the UK Biobank study has been reported in detail elsewhere (30). The polygenic risk score for hypertension was based on a recent GWAS of individuals of European ancestry (31). Therefore, the study considered only individuals who self-reported as British or other white backgrounds. We excluded SNPs that were missing from the UK Biobank study. Independent SNPs were selected based on the *P*-value by using the linkage disequilibrium (LD) clumping procedure (at *R*^2^ < 0.01) conducted in PLINK version 2.0 (https://www.cog-genomics.org/plink2). The polygenic risk score was calculated across all selected SNPs associated with hypertension totaling 118 (Supplementary Table 1) (31). For each individual in the UK Biobank sample, we calculated polygenic risk scores, defined as the sum of the number of risk alleles (0, 1, or 2) present at each locus weighted by the natural logarithm of the estimated odds ratio for that locus. Polygenic risk scores were then *z*-standardized based on values for all individuals and categorized into low (lowest quintile), intermediate (quintiles 2–4), or high (highest quintile) risk (32).

### Incident Hypertension

Data on incident hypertension in the UK Biobank were based on medical history and linked to data on hospital admissions and mortality. The linkage procedure can be found on the website (http://content.digital.nhs.uk/services) in detail. We defined participants with hypertension according to the International Classification of Diseases edition 10 (ICD-10): I10 for hypertension.

### Covariates

The covariates included in the present study were as follows: age, sex (male or female), education (degree [college/university degree] or no degree), Townsend deprivation index (TDI) (33), race (white or other), physical activity, smoking status (current, previous, or never), alcohol consumption (current, previous, or never), body mass index [BMI: was calculated by dividing an individual's weight (kg) by the square of height in meter (m)], family history of hypertension, and medical history (physician diagnosis of diabetes, depression, and cancer), obtained from the self-completed baseline questionnaire. Details of these measurements can be found on the website of the UK Biobank (www.ukbiobank.ac.uk).

### Statistical Analysis

We imputed the missing covariate values (all covariates had <3% of values missing) through multiple imputation by chained equations (34). The mean and standard deviation (SD) (continuous variables) or number and percentage (categorical variables) were used to describe the participants' baseline characteristics.

We used Cox proportional hazard regression models to test the association of sleep factors with the incident hypertension risk. The duration of follow-up was calculated as the time between the date of attendance and the date of first diagnosis, date of death, or February 28, 2017, for Scotland, and February 25, 2018, for Wales and England, whichever occurred first. Hazard ratios (HRs) with 95% confidence intervals (95% CIs) were calculated. The multivariable-adjusted models were adjusted for age, sex, education, TDI, race, physical activity, smoking status, alcohol consumption, BMI, and family history of hypertension, depression, cancer, and diabetes. The proportionality of hazards assumption was assessed using the Schoenfeld residuals and was satisfied (35). We included an interaction term in the regression model to test for the statistical interaction between the sleep pattern and genetic risk categories. In addition, adjusted population attributable fractions (PAFs) and 95% CIs were calculated to estimate the proportion of hypertension cases that theoretically would not have occurred if all participants had healthy sleep factor.

We performed subgroup analyses stratified by age (<60 or ≥60 years), sex (male or female), current smoking status (yes or no), current alcohol consumption status (yes or no), physical activity (inactive [ <400 MET-h/week] or active[≥400 MET-h/week]), BMI (non-obese [ <30 kg/m^2^] or obese [≥30 kg/m^2^]), and family history of hypertension (yes or no). In addition, we conducted several sensitivity analyses. To minimize the influence of reverse causation, we performed a sensitivity analysis by excluding participants who experienced hypertension events within the first 2 years of follow-up. Moreover, the risk of incident hypertension was investigated in sensitivity analyses using non-imputed data. All analyses were performed using R software, version 4.0.0 (R Development Core Team, Vienna, Austria). *P*-values were two-sided, with statistical significance set at <0.05.

## Results

### Baseline Characteristics

This analysis included 170,378 (mean [SD] age: 53.6 [8.0] years) participants, of whom 107,499 (63.1%) were female and 156,398 (91.8%) were white (Table 2). Participants with hypertension were more likely to be older, to have a lower level of education, to have a higher BMI, to smoke, to have higher prevalence rates of cancer, diabetes, and depression, and to have a lower healthy sleep score compared with those without incident hypertension (Table 1).

**Table 1:** Baseline characteristics of participants.

	**Overall**	**No incident hypertension**	**Incident hypertension**	***P*-value**
	**(*n* = 170,378)**	**(*n* = 163,797)**	**(*n* = 6,581)**	
Age, mean (SD), y	53.6 (8.0)	53.4 (8.0)	57.8 (7.7)	< 0.001
Female	107,499 (63.1)	103,886 (63.4)	3,613 (54.9)	< 0.001
Education				< 0.001
Degree	65,682 (38.6)	63,875 (39.0)	1,807 (27.5)	
No degree	104,696 (61.4)	99,922 (61.0)	4,774 (72.5)	
TDI	−1.4 (3.0)	−1.4 (3.0)	−0.9 (3.3)	< 0.001
BMI, mean (SD), kg/m^2^	25.99 (4.15)	25.91 (4.09)	28.04 (4.92)	< 0.001
Race				< 0.001
White	156,398 (91.8)	150,435 (91.8)	5,963 (90.6)	
Other	13,980 (8.2)	13,362 (8.2)	618 (9.4)	
Smoking status				< 0.001
Never	97,933 (57.5)	94,808 (57.9)	3,125 (47.5)	
Previous	52,692 (30.9)	50,311 (30.7)	2,381 (36.2)	
Current	19,753 (11.6)	18,678 (11.4)	1,075 (16.3)	
Alcohol consumption				< 0.001
Never	6,850 (4.0)	6,465 (3.9)	385 (5.9)	
Previous	5,568 (3.3)	5,228 (3.2)	340 (5.2)	
Current	157,960 (92.7)	152,104 (92.9)	5,856 (89.0)	
Physical activity (MET-h/week)	2,649.3 (2660.7)	2,649.5 (2654.4)	2,645. 7 (2812.8)	0.910
Family history of hypertension	66,940 (39.3)	64,416 (39.3)	2,524 (38.4)	0.116
Cancer	11,770 (6.9)	11,117 (6.8)	653 (9.9)	< 0.001
Diabetes	3,108 (1.8)	2,612 (1.6)	496 (7.5)	< 0.001
Depression	14,030 (8.2)	13,258 (8.1)	772 (11.7)	< 0.001
Healthy sleep score				< 0.001
0–1	13,072 (7.7)	12,243 (7.5)	829 (12.6)	
2	36,270 (21.3)	34,512 (21.1)	1,758 (26.7)	
3	60,895 (35.7)	58,620 (35.8)	2,275 (34.6)	
4	47,690 (28.0)	46,265 (28.2)	1,425 (21.7)	
5	12,451 (7.3)	12,157 (7.4)	294 (4.5)	
Genetic risk category				< 0.001
Low genetic risk	34,073 (20.0)	32,939 (20.1)	1,134 (17.2)	
Intermediate genetic risk	102,229 (60.0)	98,277 (60.0)	3,952 (60.1)	
High genetic risk	34,076 (20.0)	32,581 (19.9)	1,495 (22.7)	

*BMI, body mass index; SD, standard deviation; TDI, Townsend deprivation index. Data are presented as n (percent) unless otherwise indicated*.

### Associations of Sleep Factors With Incident Hypertension

During a median (interquartile range) follow-up of 9.0 (8.3–9.7) years, 6,581 incident hypertension cases were recorded. In the age- and sex-adjusted model, evening chronotype, long (≥9 h) or short (<7 h) sleep duration, insomnia, snoring, and often/always daytime sleep were significantly associated with an increased incident hypertension risk (Supplementary Table 2). In the multivariable-adjusted model, these associations remained statistically significant except for evening chronotype (Supplementary Table 2).

When these five sleep factors were reclassified as either a high (reference) or a low risk, sleep 7–9 h per day (HR, 0.86; 95% CI, 0.82–0.91), no insomnia (HR, 0.85; 95% CI, 0.80–0.91), no snoring (HR, 0.92; 95% CI, 0.88–0.97), and no frequent daytime sleepiness (HR, 0.94; 95% CI, 0.89–0.99) were independently associated with a decreased incident hypertension risk in the multivariable-adjusted model (Table 2).

**Table 2:** Risk of incident hypertension according to low-risk sleep factors.

**Low-risk sleep factors** **^b^**	***n*/*N***	**Age- and sex-adjusted**		**Multivariable-adjusted** **^a^**		**PAF (%)**
		**HR (95% CI)**	***P*-value**	**HR (95% CI)**	***P*-value**	
Sleep 7–9 h per day	4,152/120,006	0.75 (0.71–0.79)	<0.001	0.86 (0.82–0.91)	<0.001	5.2 (3.6–6.9)
No insomnia	1,363/44,400	0.82 (0.77–0.87)	<0.001	0.85 (0.80–0.91)	<0.001	11.9 (7.7–16.3)
No snoring	3,968/116,426	0.78 (0.74–0.82)	<0.001	0.92 (0.88–0.97)	0.018	3.2 (1.5–5.1)
Early chronotype	4,003/104,585	0.89 (0.85–0.94)	<0.001	0.96 (0.91–1.01)	0.092	1.6 (0.5–3.4)
No frequent daytime sleepiness	4,716/134,132	0.82 (0.77–0.87)	<0.001	0.94 (0.89–0.99)	0.027	1.7 (0.2–3.0)
All five low-risk sleep factors	294/12,451	0.68 (0.60–0.76)	<0.001	0.81 (0.71–0.89)	<0.001	18.2 (7.3–25.1)

*CI, confidence interval; HR, hazard ratio; PAF, population attributable fraction*.

a *Model was adjusted for age, sex, education, TDI, race, physical activity level, smoking status, alcohol consumption, family history of hypertension, body mass index, depression, cancer, and diabetes. All sleep factors were included simultaneously in the same model*.

b *Long (≥9 h) or short (<7 h) sleep duration, frequent daytime sleepiness, insomnia, evening chronotype, and snoring were the reference groups*.

We also calculated the PAF for each sleep factor separately and the combination of the five sleep factors (Table 2). The estimated PAFs attributable to pre-existing the low-risk sleep factors ranged from 1.6% (for chronotype) to 11.9% (for insomnia). For participants who were adherent to all five of the low-risk sleep factors, the PAF was 18.2% (95% CI: 7.3–25.1%), suggesting that 18.2% of hypertension cases in this cohort would not have occurred if all participants had been in the low-risk group for all five sleep factors.

When these five sleep factors were considered jointly by using the healthy sleep score, the risk of hypertension decreased significantly with an increasing healthy sleep score (Table 3; *P* for trend <0.001). In the multivariable-adjusted model, compared to participants with a healthy sleep score of 0–1, participants with a healthy sleep score of 2 (HR, 0.90; 95% CI, 0.83–0.98), 3 (HR, 0.81; 95% CI, 0.75–0.88), 4 (HR, 0.74; 95% CI, 0.68–0.81), or 5 (HR, 0.67; 95% CI, 0.59–0.77) had increasingly lower risks of hypertension. Each additional healthy sleep factor (per 1-point increase in score) was associated with a 9% (HR, 0.91; 95% CI, 0.89–0.93) lower risk of hypertension (Table 3). The association per 1-point higher score was similar in subgroups that were classified by sex, current smoking status, alcohol consumption status, total physical activity, BMI, or family history of hypertension (all *P* for interaction >0.05) (Supplementary Table 3). In the sensitivity analyses, the results did not markedly change after using non-imputed data (Supplementary Table 4), or excluding participants who experienced hypertension events within the first 2 years of follow-up (Supplementary Table 5). In addition, the results were not materially different for the weighted healthy sleep score (Supplementary Table 6).

**Table 3:** Association between healthy sleep score and incident hypertension.

**Healthy sleep score**	***n*/*N***	**Age- and sex-adjusted**		**Multivariable-adjusted** **^a^**	
		**HR (95% CI)**	***P*-value**	**HR (95% CI)**	***P*-value**
0–1	860/13,378	1.00 (reference)	–	1.00 (reference)	–
2	1,802/37,145	0.77 (0.71–0.83)	<0.001	0.90 (0.83–0.98)	0.013
3	2,329/62,262	0.62 (0.57–0.67)	<0.001	0.81 (0.75–0.88)	<0.001
4	1,452/48,697	0.51 (0.47–0.56)	<0.001	0.74 (0.68–0.81)	<0.001
5	299/12,722	0.44 (0.39–0.51)	<0.001	0.67 (0.59–0.77)	<0.001
Per 1-point increase in score	–	0.81 (0.80–0.83)	<0.001	0.91 (0.89–0.93)	<0.001
*P*-value for trend	–	<0.001		<0.001	

*CI, confidence interval; HR, hazard ratio*.

a *Model was adjusted for age, sex, education, TDI, race, physical activity level, smoking status, alcohol consumption, family history of hypertension, body mass index, depression, cancer, and diabetes. All sleep factors were included simultaneously in the same model*.

### Joint Association of Sleep Pattern and Genetic Risk With Incident Hypertension

**Figure 1:**
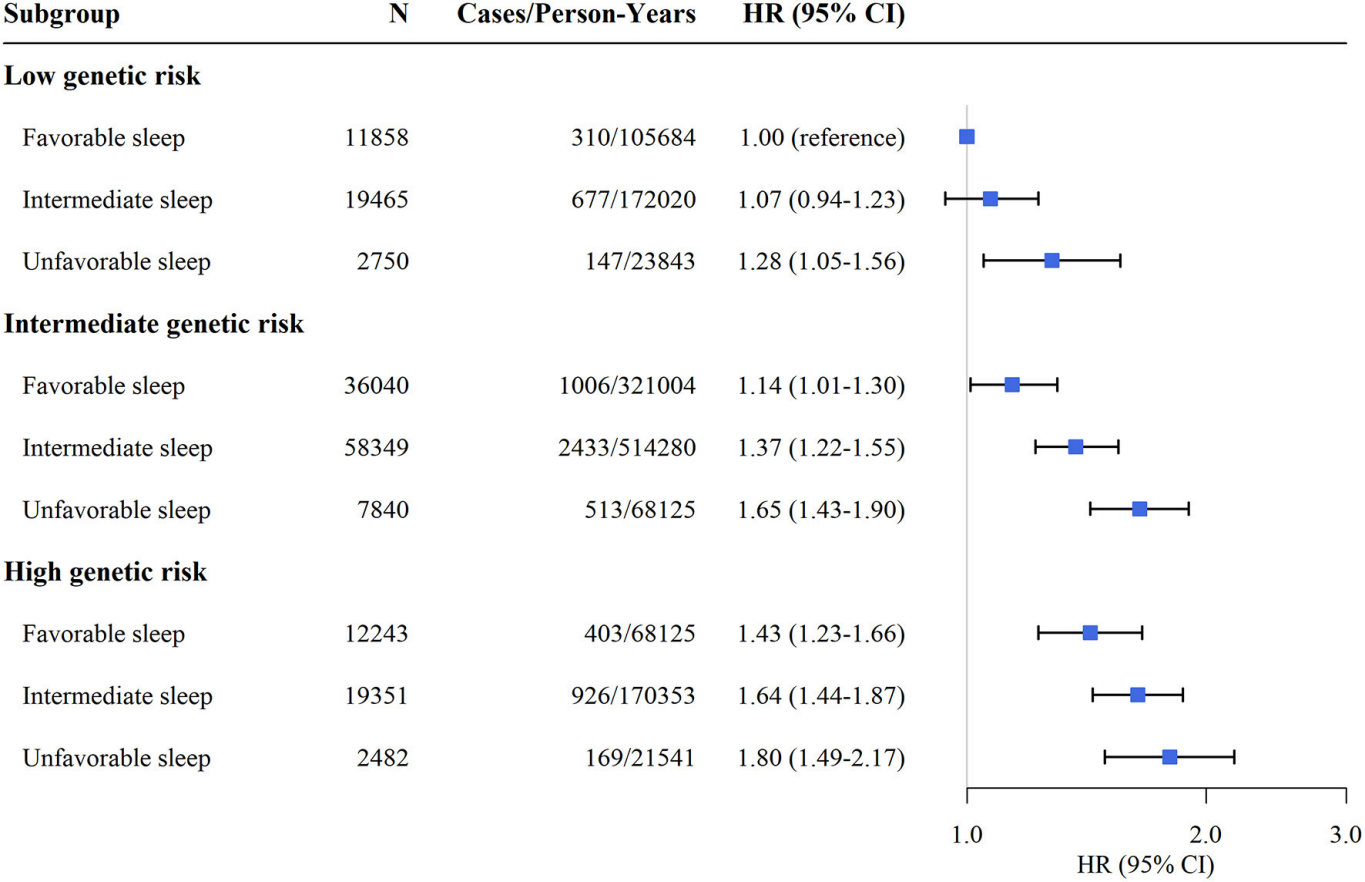
Risk of incident hypertension according to sleep pattern and genetic risk. CI, confidence interval; HR, hazard ratio. The results were obtained after adjusting for age, sex, education, TDI, race, physical activity level, smoking status, alcohol consumption, family history of hypertension, body mass index, depression, cancer, and diabetes.

Supplementary Figure 2 shows the cumulative incidence of hypertension according to sleep pattern and genetic risk. We further assessed the joint association of the healthy sleep score and polygenic risk score with the risk of hypertension. We found that participants with an unfavorable sleep pattern and high genetic risk had the highest risk of hypertension, even though there was no statistically significant interaction between the healthy sleep score and genetic susceptibility to hypertension (*P* for interaction = 0.693) (Figure 1). Participants with an unfavorable sleep pattern and high genetic risk had a 1.80-fold greater risk of hypertension (HR, 1.80; 95% CI, 1.49–2.17) than participants with a favorable sleep pattern and low genetic risk. The results were not materially different for the weighted healthy sleep score (Supplementary Table 7). Moreover, within each genetic risk stratum there was an increase in the strength of the association with a decreasing number of favorable sleep factors (*P*-value for trend <0.05) (Table 4). In the low genetic risk group, an unfavorable sleep pattern was associated with an increased risk of hypertension (HR, 1.29; 95% CI, 1.06–1.58); the association of hypertension risk with sleep pattern was similar in both the intermediate and high genetic risk groups.

**Table 4:** Association of sleep pattern with incident hypertension in genetic risk strata.

**Sleep pattern category**	**Low genetic risk**			**Intermediate genetic risk**			**High genetic risk**		
	**Favorable**	**Intermediate**	**Unfavorable**	**Favorable**	**Intermediate**	**Unfavorable**	**Favorable**	**Intermediate**	**Unfavorable**
*n*/*N*	310/11,858	677/19,465	147/2,750	1,006/36,040	2,433/58,349	513/7,840	403/12,243	923/19,351	169/2,482
HR (95% CI)^a^	1.00 (reference)	1.08 (0.94–1.24)	1.29 (1.06–1.58)	1.00 (reference)	1.19 (1.11–1.29)	1.43 (1.28–1.59)	1.00 (reference)	1.15 (1.02–1.3)	1.26 (1.05–1.52)
*P*-value	–	0.276	0.013	–	<0.001	<0.001	–	0.019	0.015
*P*-value for trend	0.021			<0.001			0.006		

*CI, confidence interval; HR, hazard ratio*.

a *Cox proportional hazards regression adjusted for adjusted for age, sex, education, TDI, race, physical activity level, smoking status, alcohol consumption, family history of hypertension, body mass index, depression, cancer, and diabetes*.

## Discussion

Using data from a large, population-based cohort study, we found that a healthy sleep score based on five potentially modifiable sleep factors, including daytime sleepiness, sleep duration, insomnia, snoring, and chronotype, was inversely associated with the future hypertension risk. Interestingly, this inverse association persisted for all subgroup analyses, including after stratification by genetic risk. Approximately 18.2% of hypertension cases could potentially be prevented if all participants had all five healthy sleep factors. These results provide evidence for the importance of healthy sleep in preventing hypertension and reinforce the tremendous potential of primary prevention.

Existing evidence, together with our result of single sleep factors revealed that healthy sleep factors, such as an early chronotype, no frequent daytime sleepiness, 7–9 h per day sleep duration, no insomnia, and no snoring were independently associated with a decreased incident hypertension risk (9, 13, 14, 36, 37). In our study, out of the five sleep factors, insomnia was component of the healthy sleep score that showed substantially the highest PAF for hypertension. It is reasonable that insomnia is more modifiable and precisely targetable through behavioral therapies (38). Therefore, future clinical trials or community-based intervention studies should be conducted to test whether sleep interventions for insomnia can reduce subsequent incident hypertension risk.

It is important to evaluate the combination of these sleep factors because they are often interconnected. In agreement with our findings, previous studies evaluating other combinations of sleep factors indicated that the combination of insomnia and short sleep duration was strongly associated with the risk of incident hypertension (13, 21). However, despite this, when examining the different combinations of sleep factors, none of the combinations were as protective as the combination of all five sleep factors. More than 18% of hypertension cases could be prevented though modification of the combined five sleep factors. Our study, the largest to date, considered the joint effect of five major sleep factors on the risk of hypertension by constructing a healthy sleep score that reflects a more comprehensive sleep pattern. The reduction in the risk of hypertension was associated with healthy sleep factors in the present study, which highlights the importance of considering sleep factors in the management of blood pressure. The healthy sleep score defined in the present study provides a significant reference for sleep management and the identification of high-risk populations.

The potential mechanism underlying the association between combined sleep factors and the risk of hypertension is not well-understood. However, these sleep factors may individually act through several mechanisms that could operate synergistically to affect the risk of hypertension. For instance, a shortened sleep duration, insomnia symptoms, or excessive daytime sleepiness possibly relates to pathways influencing sympathetic nervous system activity, which lead to blood vessel constriction, increasing blood pressure (39–41). Habitual snoring is thought to be closely related to sleep apnea; repeated apneic episodes cause oxidative stress, accelerate atherosclerosis in the coronary and intracranial arteries, and activate hemodynamics, elevating sympathetic activity and pulmonary artery pressure (42, 43). The circadian shift toward evening causes a longer-term circadian misalignment, which strengthens the association with arterial hypertension (44).

To our knowledge, the present study is the first prospective cohort study to investigate the joint association of sleep pattern and genetic risk with incident hypertension risk. We found that no statistically significant interaction between sleep pattern and genetic risk with regard to hypertension, which suggests that the effect of sleep pattern on the risk of hypertension might be independent of the genetic risk. Interestingly, we found a strong positive association between an unfavorable sleep pattern and the risk of hypertension irrespective of the prevalent genetic risk variants. Although a genetic risk for hypertension among participants from the UK Biobank has been reported previously (31), our results provide evidence that healthy sleep factors may be associated with low risk regardless of the individuals' genetic risk profile. Therefore, our study highlights the fact that adhering to a favorable sleep pattern may be greatly beneficial in the primary prevention of hypertension among the entire population. These results should be used to strengthen the importance of modifiable risk factors in the management of hypertension, as well as to convince individuals of the importance of following healthy sleep recommendations.

### Strengths and Limitations

To our knowledge, this is the first prospective cohort study to investigate the associations of five joint sleep factors with risk of incident hypertension. Another strength of this study was the large sample size, which enabled a detailed investigation of the combination of sleep factors and genetic risk. Other strengths include the comprehensive collection of sociodemographic status, medical data, and lifestyle information by self-report, which enabled us to incorporate the most prevalent lifestyle factors convincingly linked to hypertension. However, the present study also has several potential limitations. First, this is an observational study, and the associations between sleep pattern and the risk of hypertension cannot be interpreted as causal. Second, this analysis focused on only five sleep factors. Expanding the range of sleep factors (i.e., rapid eye movement sleep factor and restless legs syndrome) would be of interest in future studies. Third, although a series of confounding factors were adjusted for in the analyses, the possibility of unmeasured or unknown confounding factors may remain. Fourth, the incidence of hypertension might be underestimated by potential underdiagnosed hypertension (45). Finally, the data regarding the sleep factors in the UK Biobank were self-reported, which may have led to some misclassification.

### Conclusion

In conclusion, healthy sleep score combining daytime sleepiness, sleep duration, insomnia, snoring, and chronotype are predictive of incident hypertension. Meanwhile, unfavorable sleep pattern was associated with a higher risk of incident hypertension, regardless of genetic risk. These findings could have important implications for understanding the mechanisms underlying hypertension and provide future opportunities for early intervention.

## Data Availability Statement

Data are available in a public, open access repository. The UK Biobank data are available from the UK Biobank on request (www.ukbiobank.ac.uk/).

## Ethics Statement

The studies involving human participants were reviewed and approved by North West Multicenter Research Ethics Committee (11/NW/0382). The patients/participants provided their written informed consent to participate in this study. Written informed consent was obtained from the individual(s) for the publication of any potentially identifiable images or data included in this article.

## Author Contributions

CM, Z-HL, and Q-MH designed the research and developed the analytical plan. CM directed the study. Z-HL and Q-MH performed the statistical analyses and had primary responsibility for writing the manuscript. P-DZ, DL, DS, X-RZ, W-FZ, QC, P-LC, and W-QS contributed to data cleaning. CM, VB, XG, X-BW, and VC contributed to the analysis or interpretation of the data. All authors critically reviewed the manuscript for important intellectual content.

## Funding

This work was supported by the National Natural Science Foundation of China (Grant Number: 81973109), the Guangdong Province Universities and Colleges Pearl River Scholar Funded Scheme (Grant Number: 2019), the Construction of High-level University of Guangdong (Grant Numbers: G820332010, G618339167, G618339164, and G621339832), and the National Institutes of Health/National Institute on Aging (NIH/NIA) (Grant Number: P30AG028716). The funders played no role in the study design or implementation; data collection, management, analysis, or interpretation; manuscript preparation, review, or approval; or the decision to submit the manuscript for publication.

## Conflict of Interest

The authors declare that the research was conducted in the absence of any commercial or financial relationships that could be construed as a potential conflict of interest.

## Publisher's Note

All claims expressed in this article are solely those of the authors and do not necessarily represent those of their affiliated organizations, or those of the publisher, the editors and the reviewers. Any product that may be evaluated in this article, or claim that may be made by its manufacturer, is not guaranteed or endorsed by the publisher.
