# Supplementary material for: Healthy Sleep Associated With Lower Risk of Hypertension Regardless of Genetic Risk: A Population-Based Cohort Study
Source: Front Cardiovasc Med. 2021 Nov 18;8:769130. doi: 10.3389/fcvm.2021.769130 (PMC8637045; doi:10.3389/fcvm.2021.769130)
Supplement: Supplementary file 1 [file Data_Sheet_1.pdf]

Healthy Sleep Associated with Lower Risk of Hypertension Regardless of Genetic Risk: A Population-based Cohort study

Contents

1. eAppendix 2
2. Supplemental Tables 3
    Supplementary table 1S. Single-nucleotide Polymorphisms Used to Build the Genetic Risk Score for Hypertension 3
    Supplementary table 2S. Sleep Factors for Risk of Incident Hypertension 8
    Supplementary table 3S. Subgroups Analysis of Healthy Sleep Score with Incident Hypertension 10
    Supplementary table 4S. Multivariable-adjusted HRs (95%CIs) for Incident Hypertension After Excluding Participants with Missing Data for Covariates 12
    Supplementary table 5S. Multivariable-adjusted HRs (95%CIs) for Incident Hypertension After Excluding Participants with Hypertension within the First Two Years of Follow-up 13
    Supplementary table 6S. Multivariable-adjusted HRs (95%CIs) for Incident Hypertension by Different Healthy Sleep Score Among 385 292 Participants 14
    Supplementary table 7S. The Joint Association of Genetic Risk and Sleep Pattern with Hypertension 15
3. Supplemental Figures 16
    Supplementary figure 1S. Flow Chart of Participants Enrolment 16
    Supplementary figure 2S. Cumulative Risk of Incident Hypertension During Follow-up. 17

1. eAppendix

Assessment of Sleep factors

Sleep factors were obtained by self-report using a touchscreen questionnaire. Sleep duration was assessed with the following question: "About how many hours of sleep do you get in every 24 hours? (please include naps)". Insomnia symptoms were recorded by asking: "Do you have trouble falling asleep at night or do you wake up in the middle of the night?", with responses of "never/rarely", "sometimes", or "usually". Snoring information was obtained by asking, "Does your partner or a close relative or friend complain about your snoring?", with responses of "yes" or "no". Chronotype was assessed by the question, "Do you consider yourself to be?", with responses of "definitely a 'morning' person", "more a 'morning' than 'evening' person", "more an 'evening' than a 'morning' person", or "definitely an 'evening' person". Subjective daytime sleepiness was recorded based on the question, "How likely are you to doze off or fall asleep during the daytime when you don't mean to?" with responses of "never/rarely", "sometimes", "often", or "all of the time".

2. Supplemental Tables

Supplementary table 1S. Single-nucleotide Polymorphisms Used to Build the Genetic Risk Score for Hypertension

SNP	Chromosome	hg19 position	Reference EAF	EAF	Beta
rs880315	1	10796866	C	T	-0.475
rs17037390	1	11860843	G	A	-0.908
rs7515635	1	42408070	C	T	0.336
rs1620668	1	113023980	G	A	-0.535
rs2493134	1	230849359	C	T	-0.413
rs2586886	2	26932031	C	T	-0.404
rs1371182	2	165099215	C	T	-0.444
rs2594992	3	11360997	C	A	-0.334
rs11128722	3	14958126	G	A	-0.383
rs711737	3	27543655	C	A	0.334
rs6442101	3	48130893	C	T	0.396
rs6779380	3	169111915	C	T	-0.439
rs2291435	4	38387395	C	T	-0.378
rs1458038	4	81164723	C	T	0.659
rs17010957	4	86719165	C	T	-0.498
rs13107325	4	103188709	C	T	-0.837
rs4691707	4	156441314	G	A	-0.349
rs12656497	5	32831939	C	T	-0.487
rs10077885	5	114390121	C	A	-0.261
rs11953630	5	157845402	C	T	-0.38

rs1799945	6	26091179	G	C	-0.598
rs6919440	6	43352898	G	A	-0.337
rs1361831	6	127181089	C	T	-0.482
rs2969070	7	2512545	G	A	-0.315
rs3735533	7	27245893	C	T	-0.798
rs12705390	7	106410777	G	A	0.619
rs11556924	7	129663496	C	T	-0.336
rs2898290	8	11433909	C	T	0.377
rs10760117	9	123586737	G	T	0.334
rs6271	9	136522274	C	T	-0.567
rs12243859	10	18740632	C	T	-0.402
rs7076398	10	63533663	T	A	-0.563
rs12247028	10	75410052	G	A	-0.364
rs932764	10	95895940	G	A	-0.495
rs943037	10	104835919	C	T	-1.133
rs740746	10	115792787	G	A	0.486
rs592373	11	1890990	G	A	0.484
rs1450271	11	10356115	C	T	0.413
rs1156725	11	16307700	C	T	-0.447
rs7103648	11	47461783	G	A	-0.267
rs751984	11	61278246	C	T	0.341
rs3741378	11	65408937	C	T	-0.486
rs633185	11	100593538	G	C	0.522
rs11105354	12	90026523	G	A	0.909
rs3184504	12	111884608	C	T	0.498
rs936226	15	75069282	C	T	-0.549

rs2521501	15	91437388	T	A	-0.639
rs7213273	17	43155914	G	A	-0.413
rs17608766	17	45013271	C	T	-0.658
rs12958173	18	42141977	C	A	0.386
rs4247374	19	7252756	C	T	-0.446
rs1327235	20	10969030	G	A	-0.395
rs6026748	20	57745815	G	A	0.867
rs12627651	21	44760603	G	A	0.503
rs783621	1	42368035	G	A	0.329
rs2404715	1	57008778	T	C	0.313
rs60199046	1	59663341	G	A	0.165
rs2761436	1	207919748	T	C	-0.279
rs13403122	2	43078758	T	C	0.29
rs6434404	2	191494411	G	A	0.346
rs1250247	2	216299629	G	C	0.252
rs12630213	3	14954411	T	C	0.307
rs2178452	3	160370160	A	G	0.304
rs13104866	4	38402183	A	G	0.292
rs4292285	4	145271954	A	T	0.25
rs4475250	5	114375552	A	G	0.297
rs35410524	6	96885405	T	C	-0.359
rs10818775	9	125755571	T	C	0.333
rs34872471	10	114754071	C	T	-0.257
rs360158	11	9753601	A	G	-0.291
rs7107356	11	47676170	G	A	-0.281
rs61448762	11	48923756	A	G	0.439

rs7927515	11	76125330	A	C	-0.249
rs2289125	11	89224453	C	A	-0.207
rs7977389	12	49981722	C	T	0.322
rs10747570	12	50509937	G	A	0.302
rs3011549	13	113634937	C	A	0.347
rs2759308	15	81016227	A	G	-0.302
rs12596053	16	4946794	C	A	-0.318
rs35261357	16	75444572	T	C	-0.184
rs460105	16	89682006	C	T	0.327
rs12606620	18	42008097	T	G	0.319
rs2193635	18	43096236	T	C	-0.269
rs10427021	19	7259346	G	T	0.375
rs8105753	19	31927547	C	A	0.298
rs2014912	4	86715670	C	T	0.62
rs13359291	5	122476457	G	A	0.534
rs1563788	6	43308363	C	T	0.511
rs2493292	1	3328659	C	T	0.37
rs2270860	6	43270151	C	T	0.32
rs5219	11	17409572	C	T	0.32
rs11639856	16	24788645	T	A	-0.34
rs4823006	22	29451671	A	G	-0.26
rs35529250	4	40428091	C	T	-15.511
rs1008058	5	122435627	G	A	0.5535
rs9349379	6	12903957	G	A	0.289
rs4728142	7	128573967	G	A	-0.2416
rs34591516	8	142367087	C	T	0.6358

rs4387287	10	105677897	C	A	0.361
rs11229457	11	58207203	C	T	-0.312
rs7406910	17	46688256	C	T	-0.4563
rs3820068	1	15798197	G	A	0.367
rs10922502	1	89360158	G	A	-0.307
rs7562	2	28635740	C	T	0.182
rs13420463	2	37517566	G	A	0.244
rs55780018	2	208526140	C	T	-0.36
rs13112725	4	106911742	G	C	0.45
rs10059921	5	87514515	G	T	-0.417
rs6595838	5	127868199	G	A	0.236
rs6911827	6	22130601	C	T	0.19
rs78648104	6	50683009	C	T	-0.329
rs13238550	7	131059056	G	A	0.212
rs894344	8	135612745	G	A	-0.163
rs112184198	10	102604514	G	A	-0.532
rs6487543	12	26438189	G	A	0.279
rs9888615	14	53377540	C	T	-0.236
rs8016306	14	63928546	G	A	0.25
rs12941318	17	1333598	C	T	-0.226

Abbreviation: EAF, effect allele frequency; SNP, Single nucleotide polymorphism;

Supplementary table 2S. Sleep Factors for Risk of Incident Hypertension

Sleep factors	N	Cases	Age- and sex-adjusted		Multivariable-adjusted [a]	
			HR (95% CI)	P values	HR (95% CI)	P values
**Chronotype**						
Morning person	44 584	1854	1.00 (reference)	-	1.00 (reference)	-
Morning than evening	66 256	2246	0.91 (0.86-0.97)	0.003	0.97 (0.91-1.03)	0.317
Evening than morning	512 429	1982	1.04 (0.98-1.11)	0.219	1.06 (0.97-1.16)	0.207
Evening person	16 115	660	1.11 (1.02-1.22)	0.019	1.05 (0.98-1.12)	0.156
**Sleep duration**						
Recommended (7-9 h)	122 630	4240	1.00 (reference)	-	1.00 (reference)	-
Short (<7 h)	40 210	1913	1.27 (1.20-1.34)	< 0.001	1.15 (1.09-1.22)	< 0.001
Long (≥9 h)	11 364	589	1.34 (1.23-1.46)	< 0.001	1.13 (1.04-1.24)	0.005
**Insomnia**						
Never/rarely	137 129	4810	1.00 (reference)	-	1.00 (reference)	-
Sometimes	32 881	1662	1.16 (1.10-1.23)	< 0.001	1.13 (1.06-1.21)	< 0.001
Usually	4194	270	1.44 (1.27-1.63)	< 0.001	1.28 (1.19-1.37)	< 0.001
**Snoring**						
No	119 089	4067	1.00 (reference)	-	1.00 (reference)	-
Yes	55 115	2675	1.29 (1.23-1.36)	< 0.001	1.10 (1.05-1.16)	< 0.001

**Excessive daytime sleepiness**						
Never/rarely	45 363	1392	1.00 (reference)	-	1.00 (reference)	-
Sometimes	83 979	3155	1.16 (1.10-1.23)	< 0.001	1.06 (1.002-1.24)	0.048
Often/Always	44 862	2195	1.38 (1.28-1.48)	< 0.001	1.16 (1.037-1.32)	< 0.001

Abbreviations: HR: hazard ratio; CI: confidence interval;

[a] Model was adjusted for age, sex, education, TDI, race, physical activity level, smoking status, alcohol consumption, family history of hypertension, body mass index, depression, cancer, and diabetes. All sleep factors were included simultaneously in the same model.

Supplementary table 3S. Subgroups Analysis of Healthy Sleep Score with Incident Hypertension

Subgroup	HR[a] (95% CI)	P value	P interaction
**All**	0.91 (0.89-0.93)	< 0.001	
**Sex**			0.863
Male	0.90 (0.87-0.93)	< 0.001	
Female	0.90 (0.88-0.93)	< 0.001	
**Age**			0.016
<60 y	0.89 (0.86-0.92)	< 0.001	
≥60 y	0.92 (0.89-0.95)	< 0.001	
**Current smoking status**			0.339
No	0.91 (0.89-0.93)	< 0.001	
Yes	0.91 (0.86-0.96)	< 0.001	
**Alcohol consumption status**			0.476
No	0.91 (0.85-0.97)	< 0.001	
Yes	0.92 (0.86-0.98)	< 0.001	
**Total physical activity**			0.887
Inactive	0.90 (0.84-0.95)	< 0.001	
Active	0.91 (0.89-0.93)	< 0.001	
**BMI**			0.144
Non-obese	0.91 (0.88-0.93)	< 0.001	
Obese	0.90 (0.87-0.94)	< 0.001	
**Family history of hypertension**			0.200
No	0.92 (0.9-0.95)	< 0.001	
Yes	0.88 (0.85-0.92)	< 0.001	

Abbreviations: HR: hazard ratio; CI: confidence interval; BMI: body mass index;

Multivariable model was adjusted for age, sex, education, TDI, race, physical activity level, smoking status, alcohol consumption, family history of hypertension, body mass index, depression, cancer, and diabetes.

Supplementary table 4S. Multivariable-adjusted HRs (95%CIs) for Incident Hypertension After Excluding Participants with Missing Data for Covariates

Healthy sleep score	HR (95% CI)	P value
0-1	1.00 (reference)	-
2	0.87 (0.80-0.95)	0.005
3	0.82 (0.75-0.89)	< 0.001
4	0.74 (0.68-0.82)	< 0.001
5	0.68 (0.59-0.78)	< 0.001

Abbreviations: HR: hazard ratio; CI: confidence interval;

Multivariable model was adjusted for age, sex, education, TDI, race, physical activity level, smoking status, alcohol consumption, family history of hypertension, body mass index, depression, cancer, and diabetes.

Supplementary table 5S. Multivariable-adjusted HRs (95%CIs) for Incident Hypertension After Excluding Participants with Hypertension within the First Two Years of Follow-up

**Healthy sleep score**	**HR (95% CI)**	**P value**
0-1	1.00 (reference)	-
2	0.91 (0.84-1.00)	0.504
3	0.81 (0.74-0.89)	< 0.001
4	0.75 (0.68-0.82)	< 0.001
5	0.68 (0.59-0.79)	< 0.001

Abbreviations: HR: hazard ratio; CI: confidence interval;

Multivariable model was adjusted for age, sex, education, TDI, race, physical activity level, smoking status, alcohol consumption, family history of hypertension, body mass index, depression, cancer, and diabetes.

Supplementary table 6S. Multivariable-adjusted HRs (95%CIs) for Incident Hypertension by Different Healthy Sleep Score Among 385 292 Participants

Healthy sleep score	HR (95% CI)	P value
**Simple score (Primary analysis)**		
0-1	1.00 (reference)	-
2	0.89 (0.82-0.97)	0.005
3	0.80 (0.74-0.87)	< 0.001
4	0.73 (0.67-0.80)	< 0.001
5	0.65 (0.57-0.75)	< 0.001
**Weighted score***		
0~ <2	1.00 (reference)	-
2~ <3	0.89 (0.84-0.94)	< 0.001
3~ <4	0.81 (0.76-0.87)	< 0.001
4~ <5	0.74 (0.66-0.82)	< 0.001
5	0.72 (0.64-0.81)	< 0.001

Abbreviations: HR: hazard ratio; CI: confidence interval;

Multivariable model was adjusted for age, sex, education, TDI, race, physical activity level, smoking status, alcohol consumption, family history of hypertension, body mass index, depression, cancer, and diabetes.

* We constructed a weighted sleep score based on the 5 sleep factors by using the equation: weighted sleep score = (β1×sleep factor1 + β2×sleep factor2 +…+ β5×sleep factor5) × (5/sum of the β coefficients). This weighted score also ranges from 0 to 5 points but considers magnitudes of the adjusted relative risk for each factor in each sleep pattern as a combination of 5 factors.

Supplementary table 7S. The Joint Association of Genetic Risk and Sleep Pattern with Hypertension

Subgroup	Weighted score*	
	HR (95% CI)	P value
**Low genetic risk**		
Favorable sleep pattern	1.00 (reference)	-
Intermediate sleep pattern	1.00 (0.83-1.20)	0.980
Unfavorable sleep pattern	1.15 (0.95-1.39)	0.143
**Intermediate genetic risk**		
Favorable sleep pattern	1.03 (0.85-1.24)	0.788
Intermediate sleep pattern	1.26 (1.06-1.49)	0.083
Unfavorable sleep pattern	1.49 (1.26-1.77)	< 0.001
**High genetic risk**		
Favorable sleep pattern	1.31 (1.05-1.64)	< 0.001
Intermediate sleep pattern	1.52 (1.27-1.82)	< 0.001
Unfavorable sleep pattern	1.71 (1.42-2.06)	< 0.001

* We constructed a weighted sleep score based on the 5 sleep factors by using the equation: weighted sleep score = (β1×sleep factor1 + β2×sleep factor2 +…+ β5×sleep factor5) × (5/sum of the β coefficients). This weighted score also ranges from 0 to 5 points but considers magnitudes of the adjusted relative risk for each factor in each sleep pattern as a combination of 5 factors. The results were obtained after adjusting for age, sex, education, TDI, race, physical activity level, smoking status, alcohol consumption, family history of hypertension, body mass index, depression, cancer, and diabetes.

3. Supplemental Figures

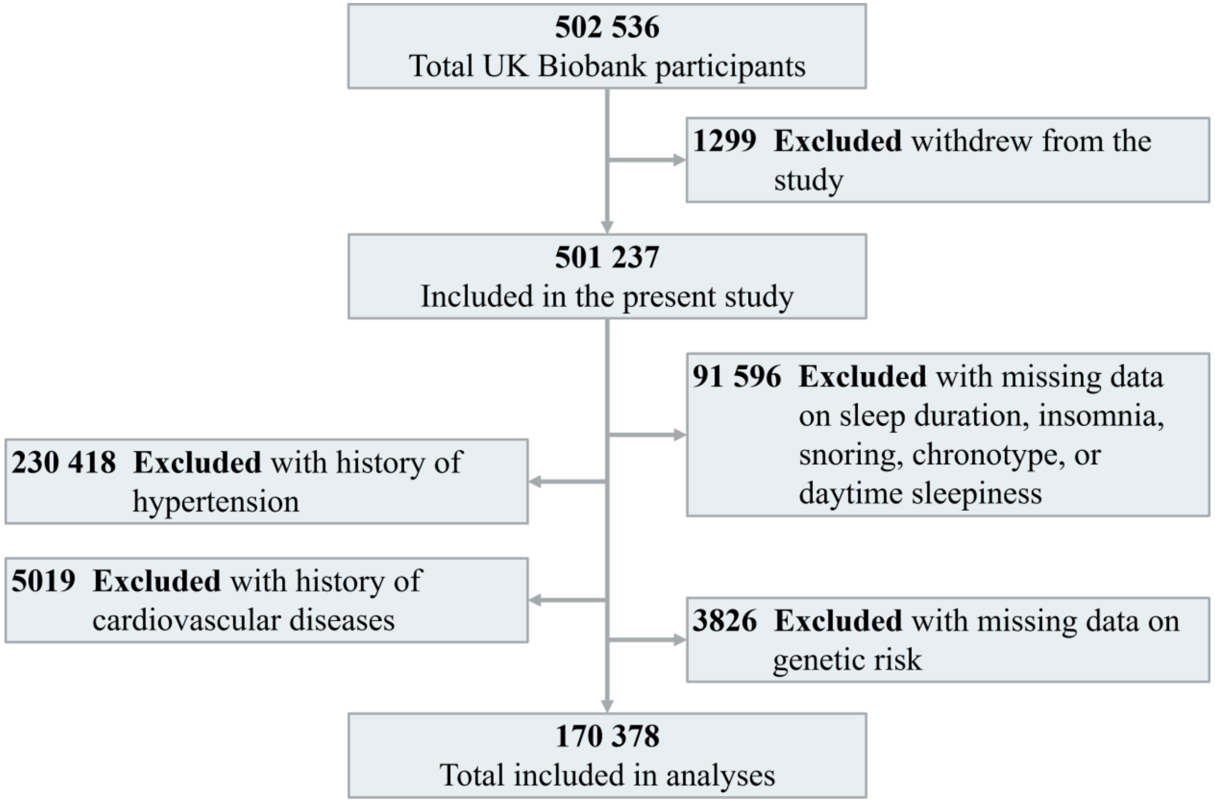

Supplementary figure 1S. Flow Chart of Participants Enrolment

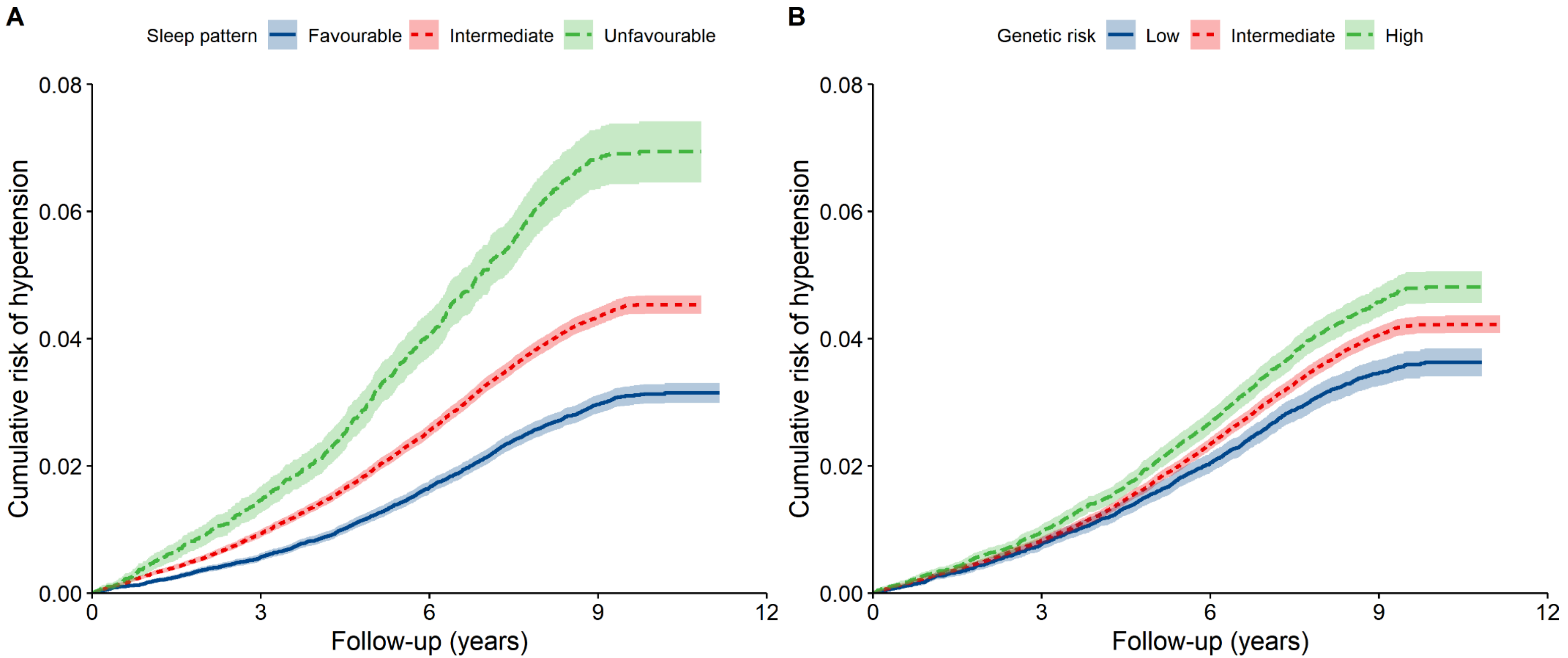

Supplementary figure 2S. Cumulative Risk of Incident Hypertension During Follow-up.
